# Experimental evidence of high tick infestation limiting chick growth and survival in a colonial seabird

**DOI:** 10.1038/s41598-024-81608-3

**Published:** 2024-12-30

**Authors:** Teresa Militão, Augustin Clessin, Amandine Gamble, José Pedro Granadeiro, Thierry Boulinier, Paulo Catry

**Affiliations:** 1 MARE - Marine and Environment Sciences Centre/ARNET - Aquatic Research Network, Ispa - Instituto Universitário, Lisboa, Portugal; 2 CEFE, UMR 5175, CNRS, EPHE, IRD, University of Montpellier, Montpellier, France; 3 École Normale Supérieure (ENS) de Lyon, Lyon, France; 4 School of Biodiversity, One Health and Veterinary Medicine, University of Glasgow, Glasgow, UK; 5 Department of Public and Ecosystem Health, Cornell University, Ithaca, USA; 6 CESAM & Departamento de Biologia Animal, Faculdade de Ciências da Universidade de Lisboa, Lisboa, Portugal

**Keywords:** Causality, Chick mortality, Ectoparasitism, Experimental field study design, Procellariiformes, Tick-induced daily blood loss, Ecology, Zoology

## Abstract

While numerous studies have established correlations between parasite load and negative effects on their hosts, establishing causality is more challenging because parasites can directly compromise host condition and survival or simply opportunistically thrive on an already weakened host. Here, we evaluated whether *Ixodes uriae*, a widespread seabird tick, can cause a decrease in growth parameters (body mass, bill length and growth rates) and survival of chicks of a colonially seabird, the black-browed albatross (*Thalassarche melanophris*) breeding on New Island (West Falkland). To investigate this, we daily removed the ticks from 28 randomly selected chicks during their first 14 days of life (treated chicks) and compared their growth and survival with 49 chicks of a control group. The tick load on control chicks correlated negatively with their body mass, growth rates and survival. Comparisons between treated and control chicks demonstrated that tick infestation depleted body mass and hampered the growth of control chicks. Treated chicks had a higher survival rate than control ones to fledgling age, but not to 14 days old, suggesting that the delayed higher mortality on control chicks was caused by the accumulation of sub-lethal effects of ticks. This study experimentally demonstrates that high tick infestation can cause reduced host growth and survival.

**Supplementary Information:**

The online version contains supplementary material available at 10.1038/s41598-024-81608-3.

## Introduction

Parasites are ubiquitous in nature and have the potential to impact various aspects of host life, including their development, reproduction, behaviour, physiology and survival^1–4^. These impacts can greatly influence host’s fitness, which ultimately may lead to significant impacts on host population dynamics^1^. Understanding how parasites influence host dynamics can be crucial for the management and conservation of wildlife populations. Parasites can deplete host resources and compromise their body condition, while also opportunistically thrive on a pre-existing weakened state of the host, making it notably difficult to unravel the causes and effects of parasitism. While numerous studies have established the correlation between parasites and their effects on hosts, establishing causality is more challenging, because it requires experimental manipulation of parasite loads and/or of the condition of the hosts (see^5–10^).

Ectoparasites, such as ticks, lice, fleas and mites, live on the surface of the host’s body. These parasites can cause a range of negative impacts on their hosts directly (e.g. by reducing parental care, body condition, and survival rate) or indirectly by increasing host susceptibility to diseases by weakening their defences and/or by infecting them with pathogens^11–13^. Seabirds are particularly vulnerable to ectoparasite infestation due to their highly social behaviour and dense breeding colonies which create ideal conditions for the transmission of these parasites among hosts. Furthermore, the high fidelity of seabirds to their breeding sites and their extended chick-rearing periods contribute to making seabirds a predictable host resource for ectoparasites, with the potential for the build-up of large local populations.

Ticks are a highly prevalent ectoparasite found on seabirds. *Ixodes uriae*is is a haematophagous ectoparasite typically found in seabird colonies in polar and sub-polar regions and is known to infest many seabird species of different orders^14^. The life cycle of this tick species is closely linked to the breeding cycle of its seabird host, with the majority of tick activity occurring during the breeding season, when they attach to the host (incubating adult or unfledged chick) and feed on their blood for 4 to 10 days^15–17^. After engorgement, ticks detach from the host to moult, in the case of larvae and nymphs, or to lay eggs, in the case of female adults^15^. Meanwhile, they remain in or around the seabird nest waiting for the host to return in the following breeding season to take the next blood meal (except adult females who die after laying the eggs). Infestation by ticks is related to several effects on their hosts, such as anaemia due to blood loss^18,19^, reduced body condition and growth rates^20,21^, increased susceptibility to diseases and infections^14^, decreased breeding performance^22^and increased mortality rates^23,24^. *I. uriae* is also known for transmitting several pathogens to its hosts, including the bacteria *Borrelia burgdorferi*, the causative agent of Lyme disease, and several viruses^14^.

Most seabird species breed in dense colonies, which are ideal habitat for ectoparasites to thrive^25^. These colonies contain abundant food resources (with numerous hosts available for the parasites to feed on) while also providing suitable habitat conditions for the parasites to conceal themselves, reproduce, and complete their life cycle. Consequently, seabirds breeding in such colonies are exposed to frequent parasitic infestations. Additionally, the large number of individual seabirds breeding simultaneously and under similar environmental conditions makes them excellent models for studying the impacts of ectoparasite infestations on wildlife populations. Among seabirds, the black-browed albatross (*Thalassarche melanophris*) is known to be frequently infested by the tick *I. uriae*, which when present in high numbers has been suggested to be responsible for increased chick mortality and low growth rates^26^. However, most studies on potential tick effects in seabirds are correlational^22,23,27–29^. To demonstrate causality, experimental approaches are required and the appropriate level of treatment needs to be carefully considered, with the difficulty that individuals within sub-colonies are not independent, especially as tick populations may vary dramatically among sub-colonies^30^. The experimental units thus need to be either individual chicks within study plots, or series of plots.

In this study, we implemented an experiment on individual chicks within study plots (sub-colonies) with the aim of assessing whether a reduced exposure to tick infestation increases body mass, size (i.e., bill length) and growth rate (i.e., daily mass gain and bill growth rate), and survival of black-browed albatross chicks. First, we ascertain whether a higher tick load (i.e., number of ticks) was correlated with poorer chick growth parameters (body mass, size and growth rate) and lower survival rate of infected chicks. Second, to establish the causality of those potential correlations, we reduced the tick load on a randomly chosen set of chicks by daily removing their ticks since the chicks hatched until they were 14 days old and compared their growth proxy parameters and survival rate with those of a control set. Given the known occurrence of hyper-infestation in black-browed albatross chicks in the study colony^26^, we anticipated detecting detrimental effects of ticks on the evaluated parameters on control birds but not on chicks whose tick load was experimentally reduced.

## Results

### Impact of daily handling on chicks

No differences were detected in body mass, bill length, daily mass gain, daily bill growth rate nor in the survival rate between the two types of control nests, i.e., between least handled control and daily handled control chicks at any age (Table 1).

**Table 1:** Regression coefficients estimates (and respective standard error, SE) and 95% credible interval of the explanatory variables used on the Bayesian linear mixed models (body mass and bill length), linear models (mass gain and bill growth rate) or generalized linear models (survival) performed to assess the associations between the tick load (i.e., number of ticks), age (5 versus 14 days old), sub-colony (B versus C), hatching date and test the influence of manipulation (least handled versus daily handled) on the different growth parameters and survival rate of black-browed albatross control chicks.

Parameter estimate ± SE [lower, upper 95% CI]	Body mass (g)	Bill length (mm)	Daily mass gain between 5 and 14 days old (g/day)	Daily bill growth rate between 5 and 14 days old (mm/day)	Survival rate to 14 days old
Bayes R^2^	0.65 ± 0.05	0.84 ± 0.02	0.32 ± 0.10	0.35 ± 0.10	0.51 ± 0.07
	[0.54, 0.72]	[0.80, 0.87]	[0.12, 0.49]	[0.14, 0.51]	[0.35, 0.61]
Intercept	256.13 ± 22.11	35.81 ± 0.42	42.16 ± 6.47	1.16 ± 0.09	1.38 ± 1.06
	[212.97, 299.73]	[35.00, 36.63]	[29.38, 54.95]	[0.99, 1.34]	[−0.51, 3.65]
Sigma	4.15 ± 0.21	0.27 ± 0.16			
	[3.67, 4.45]	[−0.08, 0.53]			
Sigma: age (14 days)	1.22 ± 0.23	0.83 ± 0.19			
	[0.85, 1.74]	[0.49, 1.24]			
Tick load	**−28.15 ± 12.72**	−0.20 ± 0.23	**−12.60 ± 4.11**	**−0.17 ± 0.06**	**−3.28 ± 0.96**
	**[−53.14**,** −3.14]**	[−0.66, 0.26]	**[−20.73**,** −4.51]**	**[−0.29**,** −0.06]**	**[−5.40**,** −1.66]**
Age (14 days)	**404.52 ± 38.31**	**10.07 ± 0.56**			
	**[328.71**,** 479.70]**	**[8.96**,** 11.18]**			
Sub-colony (C)	**78.90 ± 24.21**	0.28 ± 0.46	−4.84 ± 7.50	−0.06 ± 0.10	0.16 ± 1.29
	**[31.19**,** 126.24]**	[−0.62, 1.17]	[−19.70, 9.92]	[−0.27, 0.14]	[−2.43, 2.67]
Hatching date	**28.48 ± 11.34**	**0.51 ± 0.21**	5.02 ± 4.10	0.11 ± 0.06	**1.43 ± 0.69**
	**[6.07**,** 50.68]**	**[0.10**,** 0.93]**	[−3.08, 13.10]	[−0.00, 0.22]	**[0.20**,** 2.89]**
Manipulation treatment (least handled control)	−0.84 ± 21.11	0.21 ± 0.40	3.74 ± 6.81	−0.11 ± 0.09	1.26 ± 1.20
	[−42.22, 40.73]	[−0.57, 0.99]	[−9.77, 17.18]	[−0.29, 0.08]	[−0.94, 3.81]

Bayes R^2^ is a measure of how well the model explains the variance in the observed data. Credible intervals that do not include 0 are marked in bold. The symbol “:” represents an interaction between model parameters.

### Association between tick load and growth parameters and survival in control chicks

**Fig. 1:**
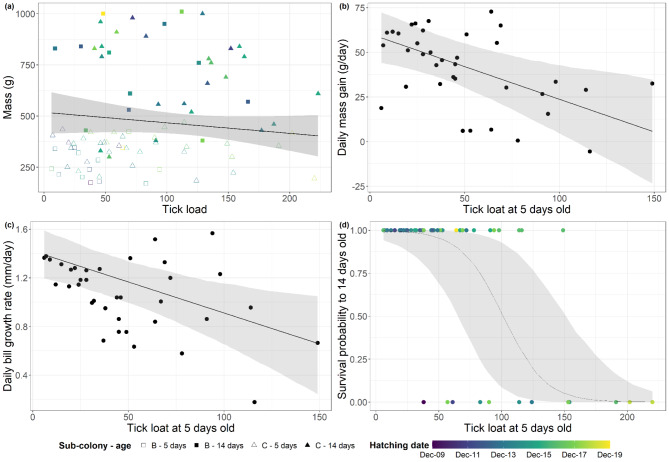
Expected values of the posterior predictive distribution of the relationships obtained from Bayesian models between the tick load on control black-browed albatross chicks at 5 days old and their (**a**) body mass at 5 and 14 days old (g), (**b**) daily mass gain between 5 and 14 days old (g/day), (**c**) daily bill growth rate between 5 and 14 days old (mm/day) and (**d**) survival probability to 14 days old. Symbols represent the observed data, black line and shade areas represent the mean and the 95% credible intervals of each response variable obtained from each model. The expected values of the posterior predictive distribution were calculated while holding constant the variables hatching date (at its mean value) and maintaining the proportion of individuals within each sub-colony, age group, and manipulation treatment. Sub-colony and age are depicted using different shaped symbols on panel (**a**), and hatching date is indicated by a colour gradient on panels (**a**), (**c**) and (**d**), as these explanatory variables also held statistical significance in the models of those response variables.

We found a negative relationship between body mass and tick load of both control chicks with 5 and 14 days old (Fig. 1a; Table 1), but no such relationship was found between tick load and bill length at those ages (Table 1). Both body mass and bill length were positively associated with the hatching date, and body mass was higher for control chicks from sub-colony C than sub-colony B (Table 1).

Control chicks with a higher tick load at 5 days old showed a reduced daily mass gain and lower bill growth rate between 5 and 14 days old (Fig. 1b and c; Table 1). No association was found between the hatching date and the daily mass gain or bill growth rate between 5 and 14 days old (Table 1). Chicks from different sub-colonies did not differ in their daily mass gain nor in their daily bill growth rate (Table 1).

The survival rate to 14 days old was higher for chicks that had a smaller tick load at 5 days old (Fig. 1d) and hatched later (Table 1), but it did not differ between sub-colonies (Table 1).

### Causal effects of experimental tick reduction

When we performed the models with the three manipulation treatment groups separately (least handled control, daily handled control and treated chicks), we did not find differences in any variable evaluated between the two types of control nests (Fig S1-S3 and Tables S1-S2). Therefore, to simplify the models, we merged the two control groups in all subsequent analyses.

**Fig. 2:**
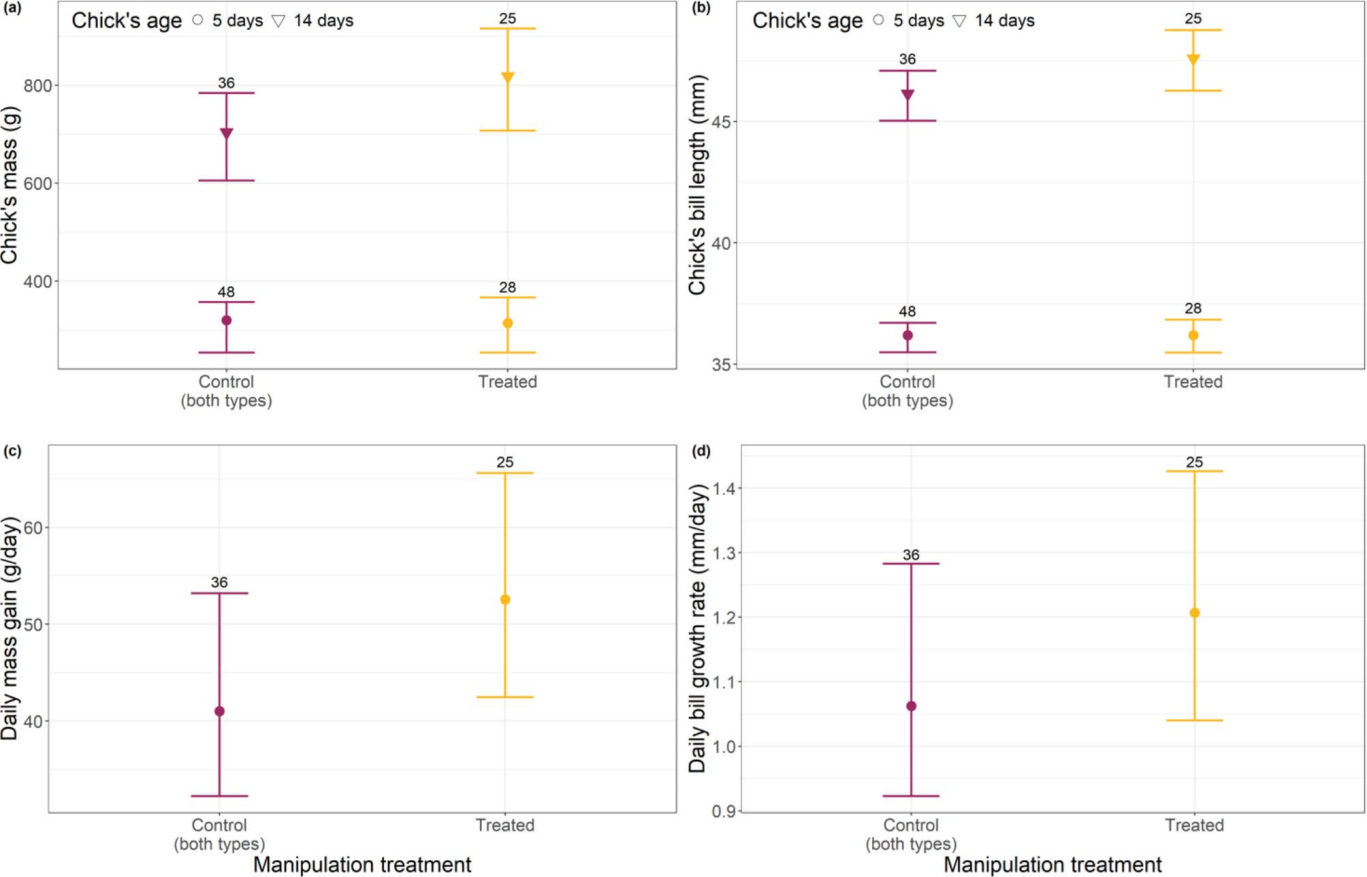
Mean (± 95% credible interval) of the expected values of the posterior predictive distribution of (**a**) chick mass (in grams) and (**b**) bill length (in millimetres) at different ages, (**c**) daily mass gain between 5 and 14 days old (g/day) and (**d**) daily mean bill growth between 5 and 14 days old (mm/day) of control and treated albatross chicks. Numbers above the error bars represent the total number of chicks of each manipulation treatment group. The expected values of the posterior predictive distribution were calculated while holding constant the variables hatching date (at its mean value) and maintaining the proportion of individuals within each sub-colony, age group, and manipulation treatment.

We found that chicks that had their ticks removed during their first 14 days of life had a greater body mass than control chicks at the end of this period, but no differences were detected at 5 nor at 59 days old (Fig. 2a; Table 2 and S2). We found no differences in bill length between control and treated chicks at any age (Fig. 2b). Treated chicks showed greater daily mass gain between 5 and 14 days old than control chicks (Fig. 2c; Table 2) and also showed a potentially slightly higher daily bill growth rate compared to control chicks (Fig. 2d; Table 2). However, the lower bound of the daily bill growth rate credible interval was 0, indicating that its difference between treated and control chicks could be very small or even negligible.

**Table 2:** Regression coefficients estimates (and respective standard error, SE) and 95% credible interval of the explanatory variables used on the Bayesian linear mixed models (body mass and bill length), linear models (mass gain and bill growth rate) or survival models performed to assess the causal effects of tick removal during the first 14 days of life (or to fledgling age) in treated black-browed albatross chicks in comparison with control chicks (both controls together).

Parameter estimate ± SE [lower, upper 95% CI]	Body mass (g)	Bill length (mm)	Daily mass gain between 5 and 14 days old (g/day)	Daily bill growth between 5 and 14 days old (mm/day)	Survival rate to 14 days old	Survival rate to fledgling
Bayes R^2^	0.72 ± 0.03	0.88 ± 0.01	0.13 ± 0.07	0.17 ± 0.07		
	[0.66, 0.77]	[0.85, 0.90]	[0.02, 0.27]	[0.04, 0.31]		
Intercept	**287.52 ± 17.27**	**36.09 ± 0.31**	44.36 ± 4.71	1.16 ± 0.06	**−12.29 ± 5.68**	**−19.67 ± 6.51**
	**[253.55**,** 321.41]**	**[35.50**,** 36.70]**	[35.09, 53.62]	[1.03, 1.28]	**[−25.17**,** −3.82]**	**[−33.60**,** −8.49]**
Sigma	**4.30 ± 0.11**	0.24 ± 0.13				
	**[4.05**,** 4.50]**	[−0.06, 0.46]				
Sigma: age 14 days	**0.98 ± 0.14**	**0.76 ± 0.15**				
	**[0.73**,** 1.27]**	**[0.49**,** 1.09]**				
Age (14 days)	**386.82 ± 34.59**	**9.96 ± 0.49**				
	**[318.64**,** 454.59]**	**[8.99**,** 10.93]**				
Sub-colony (C)	**45.44 ± 18.27**	0.12 ± 0.32	−5.18 ± 5.20	**−0.14 ± 0.07**	0.88 ± 0.60	0.36 ± 0.44
	**[9.36**,** 81.24]**	[−0.52, 0.75]	[−15.39, 5.05]	**[−0.27**,** −0.01]**	[−0.24, 2.13]	[−0.48, 1.24]
Hatching date	12.61 ± 8.41	0.29 ± 0.15	−0.19 ± 2.53	−0.01 ± 0.03	0.13 ± 0.30	0.22 ± 0.23
	[−3.91, 29.15]	[−0.00, 0.58]	[−5.14, 4.79]	[−0.08, 0.05]	[−0.46, 0.72]	[−0.24, 0.68]
Manipulation treatment (treated)	−0.04 ± 18.92	0.01 ± 0.34	**11.22 ± 5.08**	0.14 ± 0.07	−0.90 ± 0.66	**−1.02 ± 0.51**
	[−37.32, 37.27]	[−0.66, 0.67]	**[1.22**,** 21.18]**	[0.00, 0.27]	[−2.30, 0.29]	**[−2.07**,** −0.08]**
Manipulation treatment (treated):14 days old	**114.95 ± 54.31**	1.45 ± 0.78				
	**[8.21**,** 221.68]**	[−0.08, 2.98]				
Body mass at 5 days old					**−0.99 ± 0.31**	**−0.80 ± 0.24**
					**[−1.61**,** −0.40]**	**[−1.27**,** −0.34]**

Bayes R^2^ is a measure of how well the model explains the variance in the observed data. Credible intervals that do not include 0 are marked in bold. The symbol “:” represents an interaction between model parameters.

**Fig. 3:**
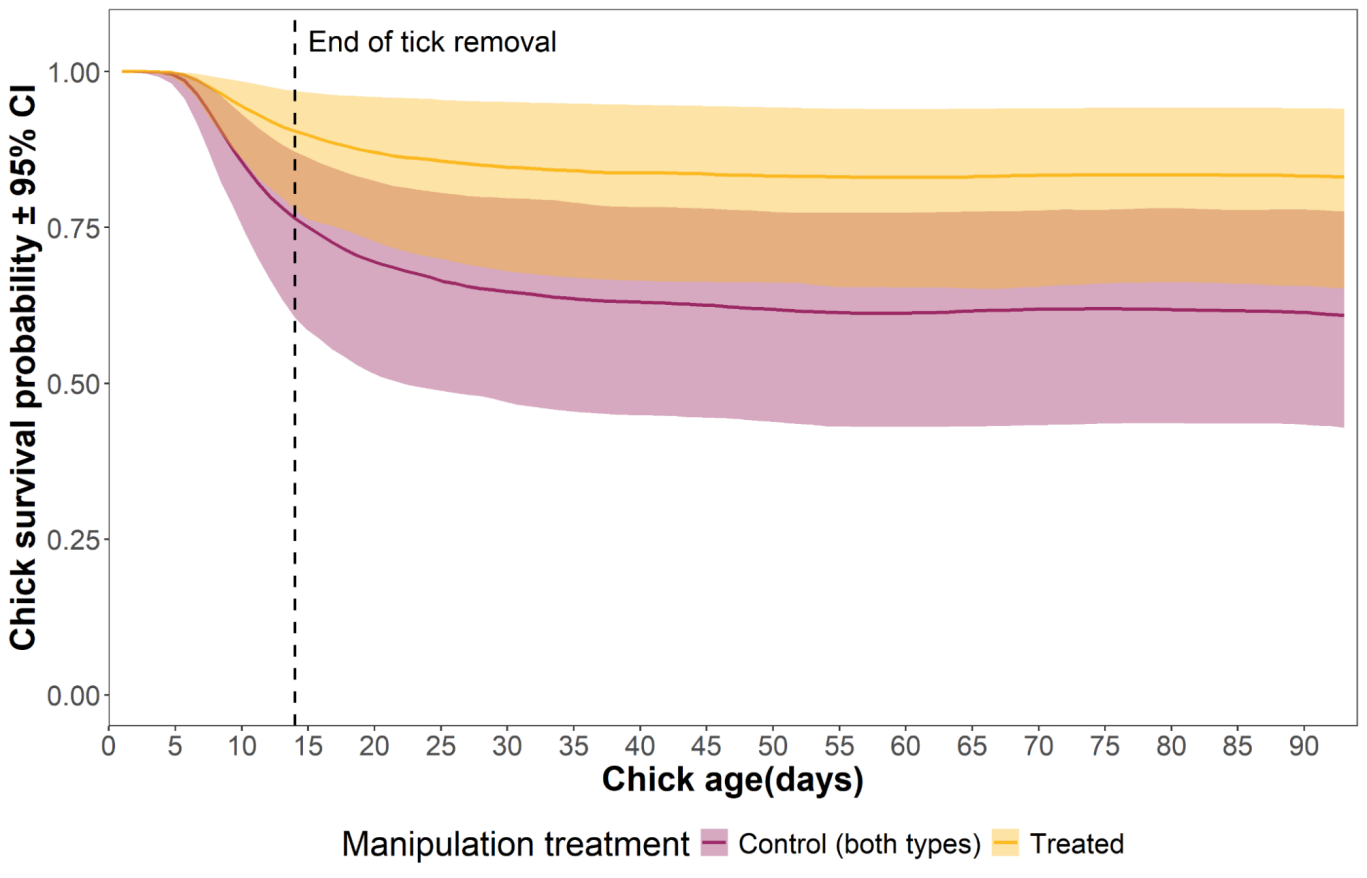
Survival probability curve of control (least handled and daily handled control chicks together) and treated albatross chicks as a function of chick age (in days) throughout the fieldwork period. Solid lines represent the mean survival probability obtained from the Bayesian model with the explanatory variables: manipulation treatment, sub-colony, hatching date and body mass at 5 days old. Shaded areas indicate the 95% credible interval obtained from that model. The vertical dashed line indicates the age at which the removal of the ticks on the treated chicks ceased (14 days old). The posterior predictive distributions were calculated while holding constant the variables hatching date and body mass at 5 days old (at their mean value) and the sub-colony to C (as this sub-colony had greater sample size than sub-colony B).

Survival probability did not differ between control and treated chicks at the end of the treatment, i.e., at 14 days old, but it was higher in treated chicks than in control ones at the end of the fieldwork season (Fig. 3; Table 2). We also found a negative relationship between body mass at 5 days old and survival probability at 14 days and at fledgling age. Based on the hazard ratio obtained, treated chicks had 0.370 times lower risk of death compared to the control chicks at the end of the fieldwork season.

## Discussion

By experimentally removing the ticks on chicks during their early development (i.e. during their first 14 days of life), we demonstrated that tick infestation had a negative effect on several developmental parameters (body mass, daily mass gain and daily bill growth rate) at 14 days old and on the survival of black-browed albatross chicks at fledgling age. Since we found no evidence of an impact of our daily handling on control chicks, the observed improvement on the growth parameters and survival in chicks subjected to the tick removal treatment can be directly attributed to the treatment itself.

In this study, we also assessed the potential negative association of tick load (i.e., the number of ticks) with the growth parameters and survival rate of control chicks. We found that body mass, daily mass gain, daily bill growth rate and survival on control chicks were negatively correlated with tick load. While some studies have also indicated similar negative correlations^21,24^, others did not detect such effects^28,29,31^.

These diverse outcomes among studies may arise from variations in susceptibility to tick-borne effects among different species and across years. For instance, guillemots (*Uria aalge*) have been found to be susceptible to the Great Island Virus carried by *I. uriae* ticks, whereas black-legged kittiwakes (*Rissa tridactyla*) were not susceptible to such infection, despite breeding sympatrically with those guillemots^32^. Even at intra-specific level, the impact of ticks might fluctuate across years, as periods of abundant food availability could empower hosts to mitigate and potentially surmount the effects of parasites. Indeed, in black-legged kittiwakes, chicks with high tick load are apparently capable of mitigating the negative effects of ticks in years of high food availability^33^.

In our study, we found a negative association between tick load and body mass, but our study was developed during the early chick-rearing period and tick load varies with chick age, frequently following a bell-shaped curve^24,34^, with much older chicks usually presenting lower tick infestation^35^. Therefore, it is important to consider the age of the chicks when comparing tick effects among studies.

The number of ticks infesting a chick and the quantity of tick-induced blood loss relative to the blood volume of the bird potentially are key factors in understanding the impact of tick infestations. Some studies reported a lack of evidence of tick effects on albatross chicks, yet the tick load in these studies was notably lower than what we found (< 18 ticks per host in other studies^28,29^contrasting with > 33 and > 68 ticks per host in the present study on control chicks at 5 and 14 days old, respectively), which implies that hosts are likely capable of overcoming tick effects at such low levels. In contrast, small chicks carrying a large number of blood-feeding ticks may lose a considerable amount of blood per day. Over time, if this blood loss is not compensated by new blood production, this may lead to anaemia and other health issues, and even death^36^. In fact, the blood loss resulting from a high tick infestation has also been identified as a potential cause of death in other seabirds, including large-bodied birds, such as adult king penguins (*Aptenodytes patagonicus*^37^).

In our study, we found no evidence of an association between tick load and bill length. This may imply that other factors beyond the scope of our study, such as parental quality^38^or chick’s sex^39^, may introduce some variability in bill length, making it challenging to identify any small effect of tick load in bill size. In contrast, we found a negative association between tick load and daily bill growth rate, but this was evaluated in a simpler model (without Nest ID random effects), suggesting that tick load may indeed affect bill length, but we were unable to detect it in a more complex model (with Nest ID random effects) given our sample size.

We found that, in comparison with control chicks, treated chicks had greater body mass at 14 days old (but not earlier), greater daily mass gain and slightly greater daily bill growth rate (between 5 and 14 days old) and greater survival rate at end of the monitoring period (but not at end of the experiment). The lack of differences in body mass between these two groups of chicks at 5 days old suggests that, despite the negative association between tick load and control chick’s body mass at this age, the benefits of tick removal may have been outweighed by the observed variations between sub-colonies and hatching dates. This is possibly because the mean tick load on control chick at 5 days old was still relatively low in contrast with the mean tick load at 14 days old (Fig. S4). Thus, given that tick effects tend to be more severe when their numbers are higher^19,40,41^ and their effects, such as blood loss, accumulate over time, it is reasonable to expect that the differences between control and treated chick would be more pronounced at 14 rather than at 5 days old.

Nonetheless, the lack of discernible benefits of tick removal at 5 days old could also be explained by the fact that pathogens, such as *B. burgdorferi*, may be mainly inoculated by ticks from the third day of feeding onwards^42^, although little evidence exist of a pathogenic effect of that bacterium in seabirds^43^. Thus, through daily tick removal, the probability of ticks inoculating pathogens on treated chicks is very unlikely, but it might also be negligible in control chicks at 5 days old, considering the recent nature of any potential inoculation.

When chicks were 59 days old, the lack of discernible differences in body mass between treated and control chicks could indicate that as chicks grow, they might surmount the impact of ticks on their body mass. However, the resemblance in body mass between treated and control chicks could also be attributed to selective mortality, whereby weaker chicks (with lower body mass) perish before reaching the age of 59 days. Indeed, we found that chicks with lower body mass at 5 days old had lower survival rate both at the end of the manipulation period (14 days old) and at fledgling age. This is similar to what was found in Magellanic penguins (*Spheniscus magellanicus*), in which the variation on body mass among chicks decreased throughout the chick-rearing until converging on a similar mass before fledging due to strong selective mortality^44^.

In contrast to the short-term effects of tick removal on body mass growth parameters that were detected on chicks at 14 days old, a greater survival rate of treated chicks compared to control ones was only detected several days after the treatment concluded, despite the selective mortality of control chicks at younger ages. A similar delayed effect of ticks on survival rates was observed in roseate tern (*Sterna dougallii*) chicks in which tick infestation during the chick’s growth period (0–23 days) negatively affected nestling survival probability^45^. This implies that the combined and accumulating sub-lethal effects of tick infestation during the brooding period may lead to deferred mortality of control chicks. Furthermore, if a chick became infected with a tick-borne disease during the treatment period (i.e., the first 14 days of life), it is possible that this infection might only reach a lethal stage later on, given the typical incubation period of most tick-borne diseases^46^.

In this study, we implemented an experimental design in which chicks were the experimental units, as the treatment (tick removal) was applied to randomly selected individual chicks. While this experimental design was time-consuming, it effectively established the causal effects of the presence of ticks on albatross chicks. The results that can be obtained with this approach outperform the ones that can be achieved by less time-consuming experiments in which a treatment, such as acaricide, is applied to a group of nests (plot-based experiment). In a plot-based experiment, establishing the causal effects of ticks is more challenging as chicks within plots are not independent and there can be confounding factors associated with the spatial distribution of plots, forcing the establishment of multiple treated and control plots to distinguish the effect of the parasite from those of the confounding factors^47,48^. Additionally, anti-parasite treatments may eliminate other parasites, not just the one under study, thereby complicating the assessment of the effects of the target parasite^47,48^. Therefore, our experimental design was well-suited for assessing tick causal effects on albatross chicks within natural infection levels.

Overall, we demonstrated experimentally that tick infestation of albatross chicks was the cause of the sub-lethal and lethal effects, rather than ticks simply taking advantage of an already weakened state of the host. These effects may have been caused directly by the ticks, due for example to the high amount of tick-induced daily blood loss and/or pathogen inoculation, but they can also be caused indirectly by weakening the immune system of the chicks making them more vulnerable to diseases/pathogens. Future studies could complement this kind of experimental study with an assessment of antibody level against potentially tick-borne agents to better evaluate the importance of direct and indirect mechanisms by which ticks affect chick fitness. In the current context of global change, the potential direct and indirect effects of ticks and tick-borne agents in widely distributed seabirds is clearly of interest to further explore in comparable systems^49^.

## Methods

### Study area and study species

This study was carried out in two sub-colonies (B and C) of black-browed albatrosses breeding at the Settlement Rookery (51°43’S, 61°19’W) on New Island (West Falkland), during the 2022/2023 breeding season. Black-browed albatrosses lay a single egg in early October in a pillar-shaped nest made from mud, guano and other surrounding materials, such as tussock leaves (*Poa flabellate*), incorporated into its structure. The egg is incubated by both parents for about 68–71 days^50^, after which the chick hatch from the second week of December onwards. After a brooding period shared by both parents of about 20 days^51^, during which chick predation is unlikely, the chick is left alone in the nest until it fledges at the end of April (after about 116 days^50^).

### Experimental design

**Fig. 4:**
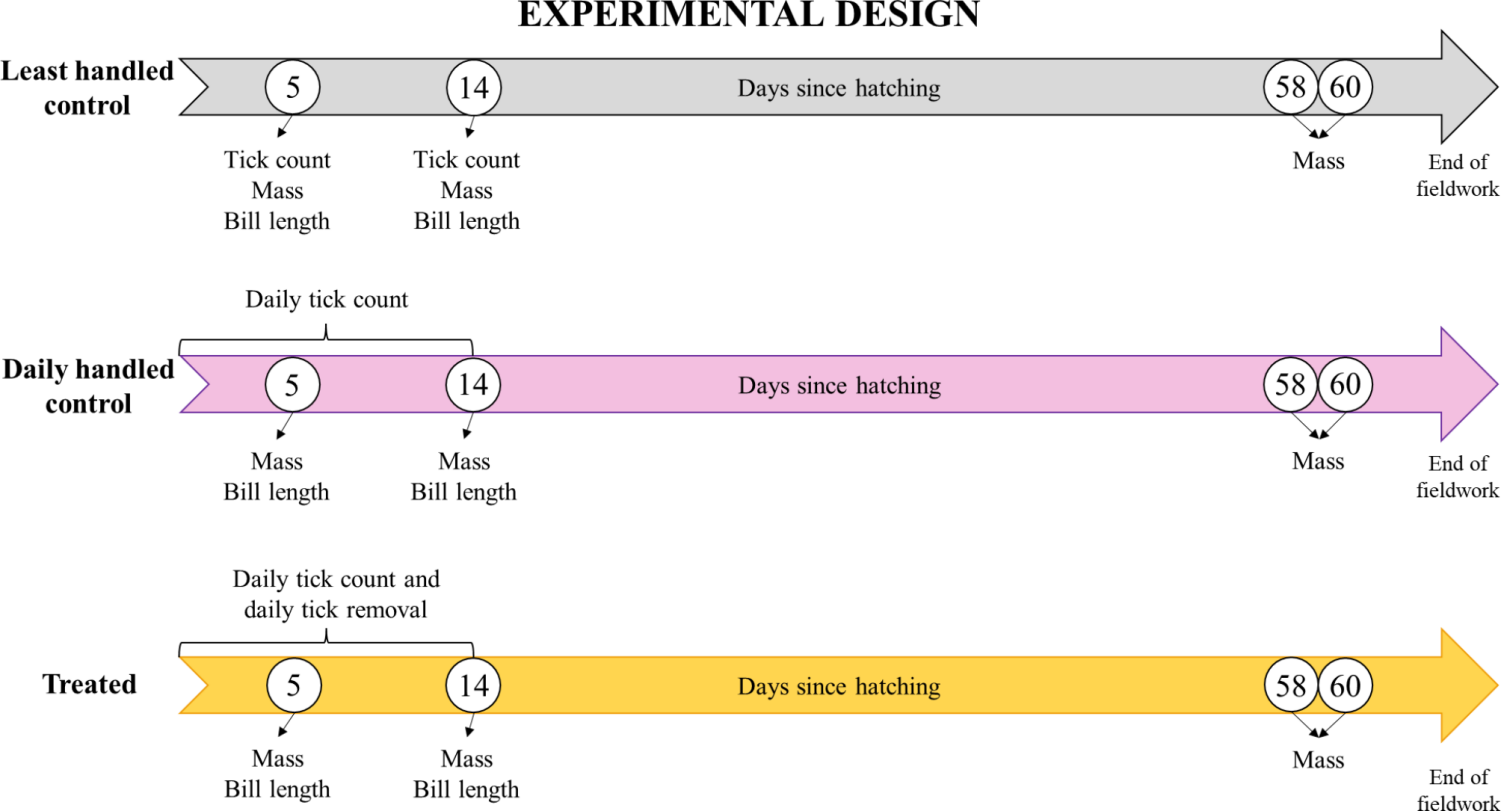
Schematic diagram of the experimental design. The white circles represent chick’s age (in days) at which sampling procedures were performed.

During the 2022/2023 breeding season, 36 and 111 active nests of the sub-colonies B and C, respectively, were visited daily (except due to extreme weather conditions) from the 9^th^ of December until the 13^th^ of March to visually determine the hatching date and chick survival. Immediately upon hatching, we randomly assigned a subset of chicks to one of the three manipulation treatment groups: (a) least handled control, (b) daily handled control and (c) treated (Table 3; Fig. 4). The chicks were assigned to these manipulation treatment groups as they hatched in a repeating pattern to achieve a good distribution of the treatment groups across hatch order and sub-colonies. In Figures S5 and S6, it is possible to visualize the random geographic distribution of the study nests in each sub-colony.

**Table 3:** Number of handled black-browed albatross chicks segregated by sub-colony, treatment group and age, and mean tick load ± standard error [minimum and maximum values] at 5 and 14 days old.

Sub-colony	Treatment group	At hatching	5 days old		14 days old		59 days old
		Chicks	Chicks	Tick load	Chicks	Tick load	Chicks
B	Least handled control	5	5	33.0 ± 11.0 [9–69]	5	68.4 ± 18.1 [9–112]	3
	Daily handled control	9	9	46.7 ± 9.5 [6–94]	7	86.0 ± 20.4 [30–166]	5
	Treated	12	12	16.8 ± 5.6 [0–55]	10	46.2 ± 15.6 [4–173]	9
C	Least handled control	19	19	80.9 ± 11.2 [22–191]	14	101.7 ± 15.3 [41–223]	11
	Daily handled control	16	15	77.5 ± 17.5 [7–220]	10	122.9 ± 14.2 [53–187]	9
	Treated	16	16	29.6 ± 6.7 [3–91]	15	52.0 ± 8.1 [6–131]	14

To assess the impact of tick infestation on the albatross chick growth parameters and survival rate, we conducted the following procedure across the three manipulation treatment groups:

- Least handled control chicks: we kept their handling to a minimum (maximum four times) by only counting their ticks, weighing them and measuring their bill length at ages of 5 and 14 days old and weighted them again before fledging at 58 and 60 days old;
- Daily handled control chicks: we conducted daily tick counts on their first 14 days of life, weighed them at 5, 14, 58 and 60 days old and measured their bill length at 5 and 14 days old;
- Treated chicks: we performed the same protocol as the daily handled control chicks, but ticks were removed daily during their first 14 days of life.

We counted all the ticks (adult females, nymphs and larvae) attached to the chicks by examining their whole body both visually and by palpation. Photographs of most typical areas where high number of ticks were found on the study chicks are presented on Fig. S7. On treated chicks, all ticks found were carefully removed by gently pulling each tick from its mouthparts with tweezers and eliminated them to avoid ticks to infest again the chicks. The chicks were weighed using a ©Pesola (300–500 g, with a precision of 0.3%, at 5 days old depending on the weight of the chick and 5 kg, with a precision of 0.3%, at 14 days old) and bill length (length from the tip of the bill until the insertion of the feathers at the bill base) was measured using a calliper of 150 mm, with a precision of 0.05 mm. At 58 and 60 days old, we weighed the chicks with a ©Pesola of 5 kg to estimate an average weight at 59 days old. Between each chick, all the material was disinfected with 0.5% Virkon solution and we also disinfected our hands with hydrogel to avoid potential propagation of any disease among chicks caused by our manipulation.

We did not count the number of ticks at 58 nor 60 days old due to the size of the chick and the development of feathers, which would hinder accurate counting. Nevertheless, no ticks were observed in featherless areas such as the bill or the feet. Furthermore, due to logistic limitations, we were not able to measure the bill length at 58 nor 60 days old.

Tick counting and biometric measures of chicks at 5 and 14 days were performed by the same person (TM) to avoid potential differences between measurers.

### Statistical analyses

All statistical analyses described below were carried out using R 4.2.1^52^ with the level of significance set to *p*< 0.05 for frequentist analyses. For Bayesian inference analyses, we considered that, given the data, a specific parameter had an effect on the response variable if the 95% credible interval of that parameter did not include the value zero^53^.

Unless otherwise specified, all Bayesian inference analysis were performed using the *brm*function from the “brms” package^54–56^, with the default (non-informative) priors, a Gaussian family, 5 chains with 60,000 iterations per chain (30,000 of which were used as warm-up), an argument *adapt_delta* of 0.99 and *max_treedepth*of 12. We assess model convergence by checking Ȓ (R-hat) values (Gelman-Rubin diagnostic), which were equal to 1.00 for all parameters of all models, indicating a good model convergence, and by visually inspecting the Markov Chain Monte Carlo (MCMC) chains^57^. We evaluated model fit by comparing the distribution of the observed response variable to 1,000 simulated datasets generated from the posterior predictive distribution using the *pp_check*function from the “brms” package^54–56^. We verified that both the Bulk and Tail Effective Sample Sizes (ESS) were greater than 10,000 for all model parameters^57^. We used the *pairs*function from the “brms” package^54–56^ to visually inspect that there was no collinearity in explanatory variables (both fixed and random effects). The posterior distribution were obtained using the *posterior_epred* function from the “brms” package^54–56^.

The sub-colony (B vs. C) to which chicks belong and hatching date (as Julian day) of each chick were incorporated to all models to account for additional potential sources of variation in body mass, bill length, growth rates and survival rate.

### Impact of daily handling and potential effects of tick load on the host

To evaluate the impact of daily handling on chick body mass and size (using bill length as a proxy of chick size) and determine whether these biometric measurements were negatively associated to tick load (i.e., number of ticks), we performed a Bayesian multivariate linear mixed model. Both body mass and bill length of least and daily handled control chicks at 5 and 14 days old were included as response variables, allowing for the correlation between these biometric measurements. This model included as explanatory variables the handling group (least handled vs. daily handled control), the tick load at each age, the age (5 vs. 14 days old), the sub-colony (B vs. C) and hatching date (as Julian day). Tick load and hatching date were standardized by subtracting the mean and dividing by the standard deviation of these variables. To take into account the pseudoreplication of the repeated measures of the same chick at different ages, we included Nest ID as random intercept. Furthermore, the residual standard deviations (σ - sigma) were modelled as a function of age to take into account for potential differences in variability across age groups (heteroscedasticity). Due to the complexity of the model, we increased the *adapt_delta* argument of the *brm* function to 0.999 to avoid divergent transitions.

Chick growth rates can also be affected by both the stress of the daily handling and the potential adverse effects of tick load. To assess these potential effects, we calculated the daily mass gain and daily bill growth rates of the chicks between the ages of 5 to 14 days old. Since mass and bill length in albatrosses species is exponential until later ages^58,59^, these rates were obtained by subtracting the body mass (or bill length) at 14 days and 5 days and then dividing by the number of days between these ages. We performed a Bayesian multivariate linear model with both daily mass gain and daily bill growth rate as response variables, allowing for the correlation between these growth rates. This model included as explanatory variables the handling group (least handled vs. daily handled control), the standardized tick load at 5 days old, the sub-colony (B vs. C) and standardized hatching date (as Julian day).

To understand if daily handling and/or tick load were negatively associated with the survival rate of albatross chicks, we contrasted the survival rate of least handled control and daily handled control chicks to 14 days old and assess if it was negatively correlated with the tick load at 5 days old. For that, we constructed a Bayesian generalised linear model, with survival rate as a binary response variable (0 represented a chick that died between the ages of 5 to 14 days old, while 1 indicated a chick that was alive at 14 days old). This model was performed using the Bernoulli family and logit link and included as explanatory variables the handling group (least handled vs daily handled control), the standardized tick load at 5 days old, the sub-colony (B vs C) and standardized hatching date (as Julian day). The posterior distribution were obtained using the *add_epred_draws *function from the “tidybayes” package^60^.

### Causal effects of experimental tick reduction

To empirically ascertain the effect of tick removal on chick body mass and bill length, we compared the values of these biometric measurements between treated (i.e., those to which ticks were experimentally removed during the first 14 days of life), least handled control and daily handled control chicks. For that, we performed a Bayesian multivariate linear mixed model with both body mass and bill length at 5 and 14 days old as response variables, allowing for the correlation between these biometric measurements. This model included manipulation treatment (treated, least handled control and daily handled control), the age (5 versus 14 days old), sub-colony (B vs. C) and standardized hatching date (as Julian day) as explanatory variables. We also included an interaction between manipulation treatment and chick age, as the benefit of tick removal may be greater at 14 than at 5 days old chicks, given that the number of ticks on control chicks increased with age (Fig. S4), which could also lead to greater differences between manipulation treatment groups at 14 than 5 days old. To take into account the pseudoreplication of the repeated measures of the same chick at different ages, we included Nest ID as random intercept. Furthermore, the residual standard deviations (σ - sigma) were modelled as a function of age to take into account for potential differences in variability across age groups (heteroscedasticity). Due to the complexity of the model, we increased the *adapt_delta* argument to 0.999 to avoid divergent transitions. As this model showed no differences in both the body mass and bill length between the two control groups (Fig. S1 and Table S1), we combined them and performed the final analysis comparing treated chicks to the merged control group. As we were not able to measure the bill length at 59 days old, the comparison of the body mass at this age between treatment groups was performed separately using a Bayesian linear model, with mass at 59 days as the response variable. This model included manipulation treatment (treated, least handled control and daily handled control), sub-colony (B vs. C) and standardized hatching date (as Julian day) as explanatory variables. As this model showed no differences body mass at 59 days old between the two control groups (Table S2), we combined them and performed the final analysis comparing treated chicks to the merged control group.

To assess whether removing ticks on the treated chicks could lead them to have greater growth rates than control chicks, we compared their daily mass gain and daily bill growth rate by using a Bayesian multivariate linear model, with these both growth rates as response variables. This model included as explanatory variables the manipulation treatment (treated, least handled and daily handled control chicks), the sub-colony (B vs. C) and standardized hatching date (as Julian day). As this model showed no differences in any of the growth rates between the two control groups (Fig. S1 and Table S1), we combined them and performed the final analysis comparing treated chicks to the merged control group.

To evaluate the effect of tick removal on chick survival, we conducted a Bayesian survival analysis where we compared the survival rates of treated, least handled and daily handled control chicks. This analysis was performed using the function *stan_surv* from the package “rstanarm”^61,62^, with the default (non-informative) priors and with the same number of chain, iterations per chain, *adapt_delta* and *max_treedepth* as described previously at beginning of the statistical analysis section. We performed two models set one to test for differences between survival rates of treated, least handled and daily handled control chicks to 14 days old and another to the last monitoring day, to understand if the effects of tick removal were detected only at end of the experiment and/or at end of the fieldwork period. Our data were right censored (Fig. S2), i.e., we knew that whether a chick was alive until the last nest monitoring visit (or until the end of the experiment, depending on the model), but we did not know if it survived after that. Therefore, our response variable was the age (in days) of death of the chick or the age at the last nest monitoring visit if the chick was alive until the end of the experiment or fieldwork season (depending on the model). We included as explanatory variables the manipulation treatment (treated, least handled and daily handled control chicks), the sub-colony (B vs. C) and standardized hatching date (as Julian day). Additionally, we also included the mass at 5 days old as a fixed factor (after standardizing it) to assess for potential selective mortality of chicks with lower mass. We chose the mass at this age, as later those individuals with lower mass could have already died which could biased the results. In Bayesian survival analysis, we need to specify the baseline hazard distribution which defines the underlying risk pattern over time. Therefore, for each model set (end of experiment and end of fieldwork period), we fitted five models with different baseline hazard distribution, namely, exponential, Weibull, Gompertz, cubic M-splines and cubic B-splines. We compared these models through effective approximate leave-one-out cross validation using the function *loo_compare *from the “loo” package^63^ and by checking the model fit using the function *ps_check *from the package “rstanarm”^61,62^. As both the lowest effective approximate leave-one-out cross validation and model fit indicated that the best model was the one performed using cubic B-spline, this was the baseline hazard distribution selected for the final models. Furthermore, the final models were performed with the two control groups merged as no differences in survival analysis were found between the two control groups (Fig. S3 and Table S1). Model convergence and EES of the final models were assessed as described above for *brm* models. The curve of the survival probability until the end of the fieldwork period was constructed based on the posterior distribution obtained using the function *posterior_survfit* from the package “rstanarm”^61,62^. With these survival models we also obtained the hazard ratio for the treated chicks. A hazard ratio of 1 indicates that tick removal did not affect the survival rate of the chicks, while a hazard ratio below or above 1 suggests that tick removal either increased or decreased the survival probability, respectively, in comparison with control chicks.

## Electronic supplementary material

Below is the link to the electronic supplementary material.

Supplementary Material 1

## Data Availability

Data from the manuscript is available at https://zenodo.org/records/14288530 (DOI: 10.5281/zenodo.14288530) or upon request to the corresponding author.
