# Supplementary material for: Experimental evidence of high tick infestation limiting chick growth and survival in a colonial seabird
Source: Sci Rep. 2024 Dec 30;14:31694. doi: 10.1038/s41598-024-81608-3 (PMC11686276; doi:10.1038/s41598-024-81608-3)
Supplement: Supplementary file 1 — Supplementary Material 1 [file 41598_2024_81608_MOESM1_ESM.docx]

SUPPLEMENTARY MATERIAL

of

**Experimental evidence of high tick infestation limiting chick growth and survival in a colonial seabird**


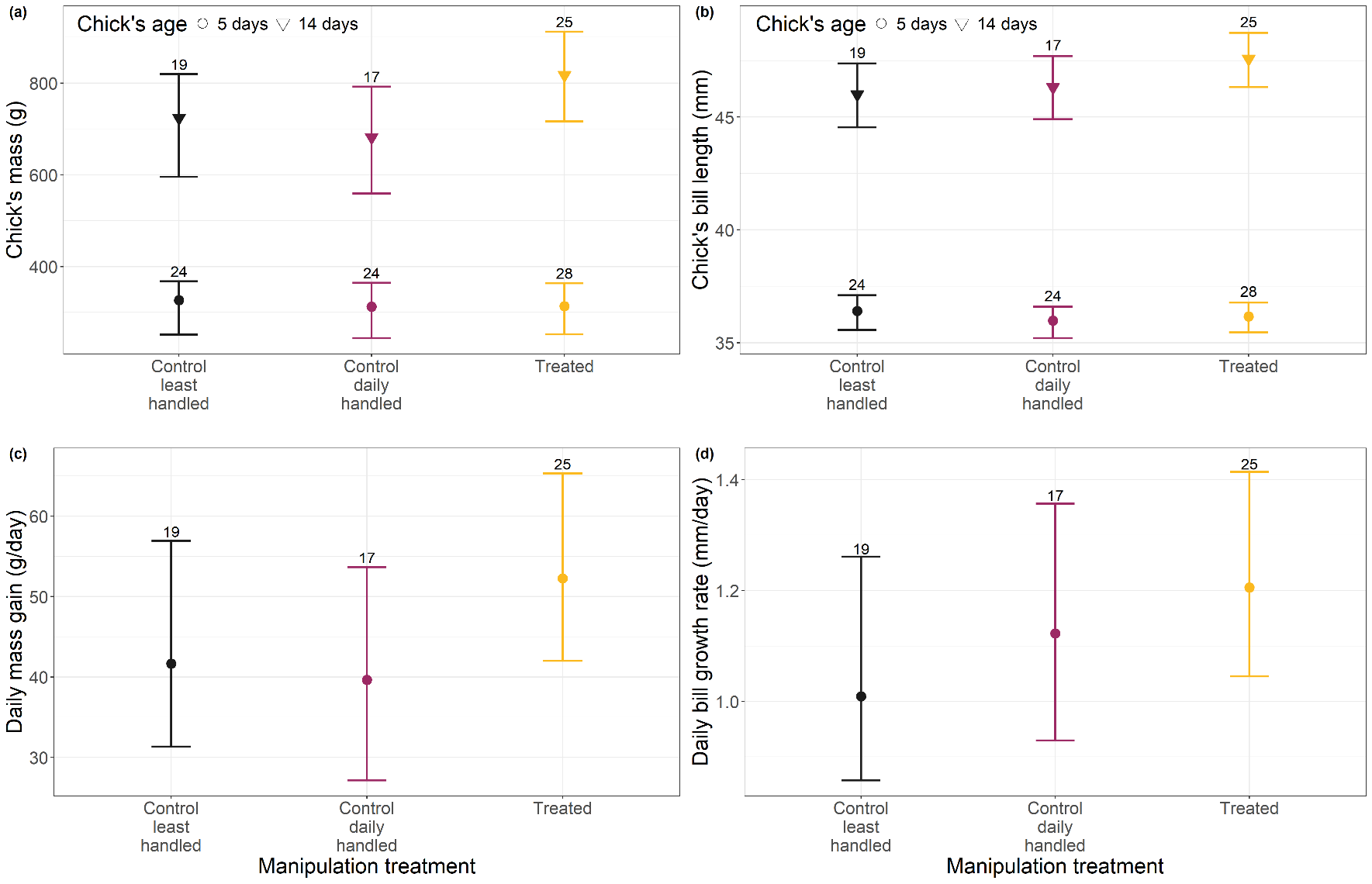


Figure S1 – Mean and 95% credible intervals of the expected posterior distribution of (a) the body mass (in grams), (b) bill length (in millimetres), (c) daily mass gain (in grams per day) and (d) daily bill growth segregated by the three manipulation treatment chicks (least handled control, daily handled control and treated chicks). Numbers above the error bars represent the total number of chicks of each manipulation treatment group. The expected values of the posterior predictive distribution were calculated while holding constant the variables hatching date (at its mean value) and maintaining the proportion of individuals within each sub-colony, age group, and manipulation treatment.


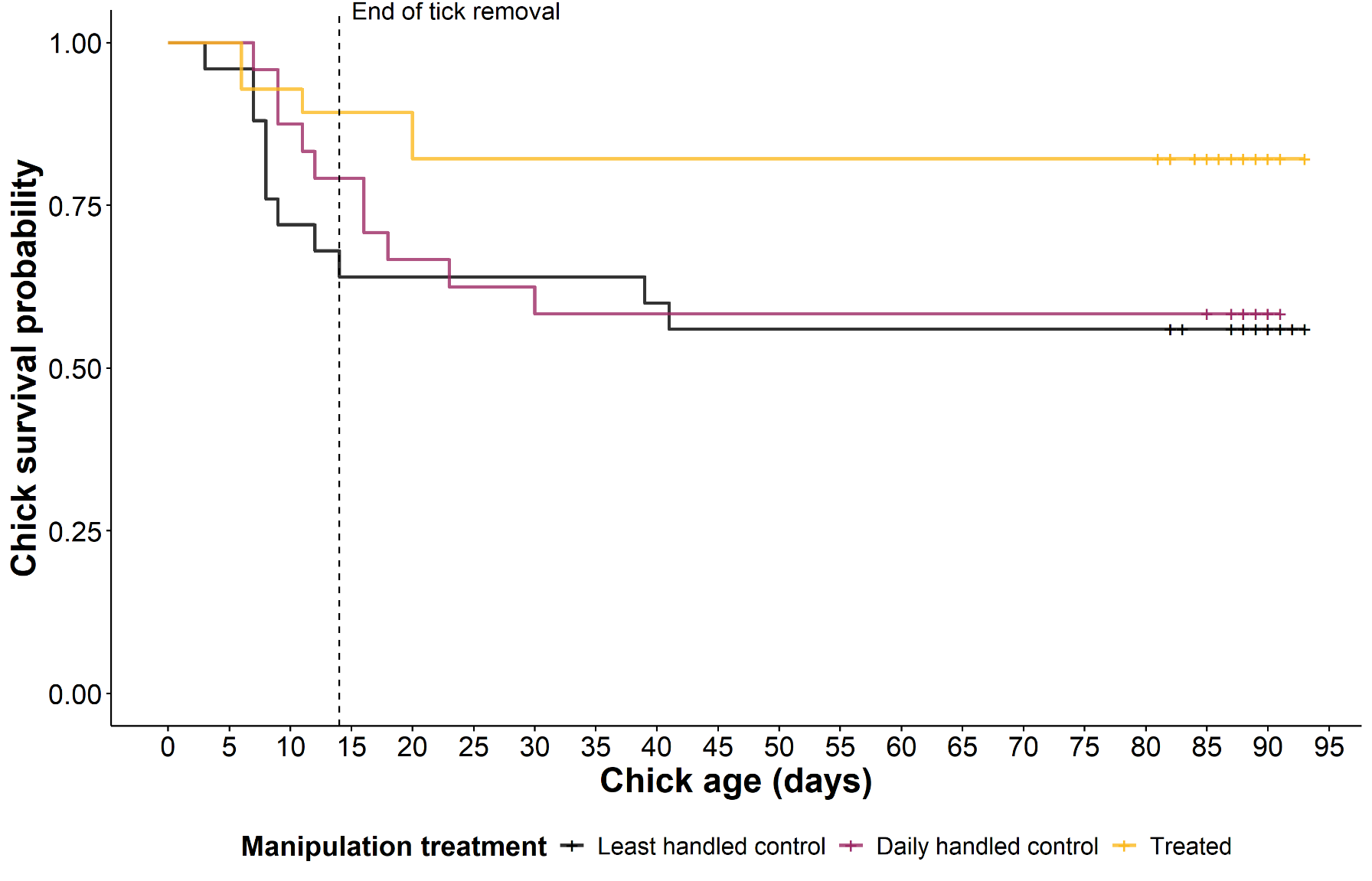


Figure S2 - Kaplan-Meier survival curve showing the probability of chick survival over time (in days) for the different manipulation treatments (least handled control, daily handled control and treated chicks). The vertical dashed line indicates the age at which the removal of the ticks on the treated chicks ceased (14 days old). The age of censoring, i.e., the age at which chicks were still alive at the last nest monitoring visit, is denoted by the plus symbols (+) along the survival curves.


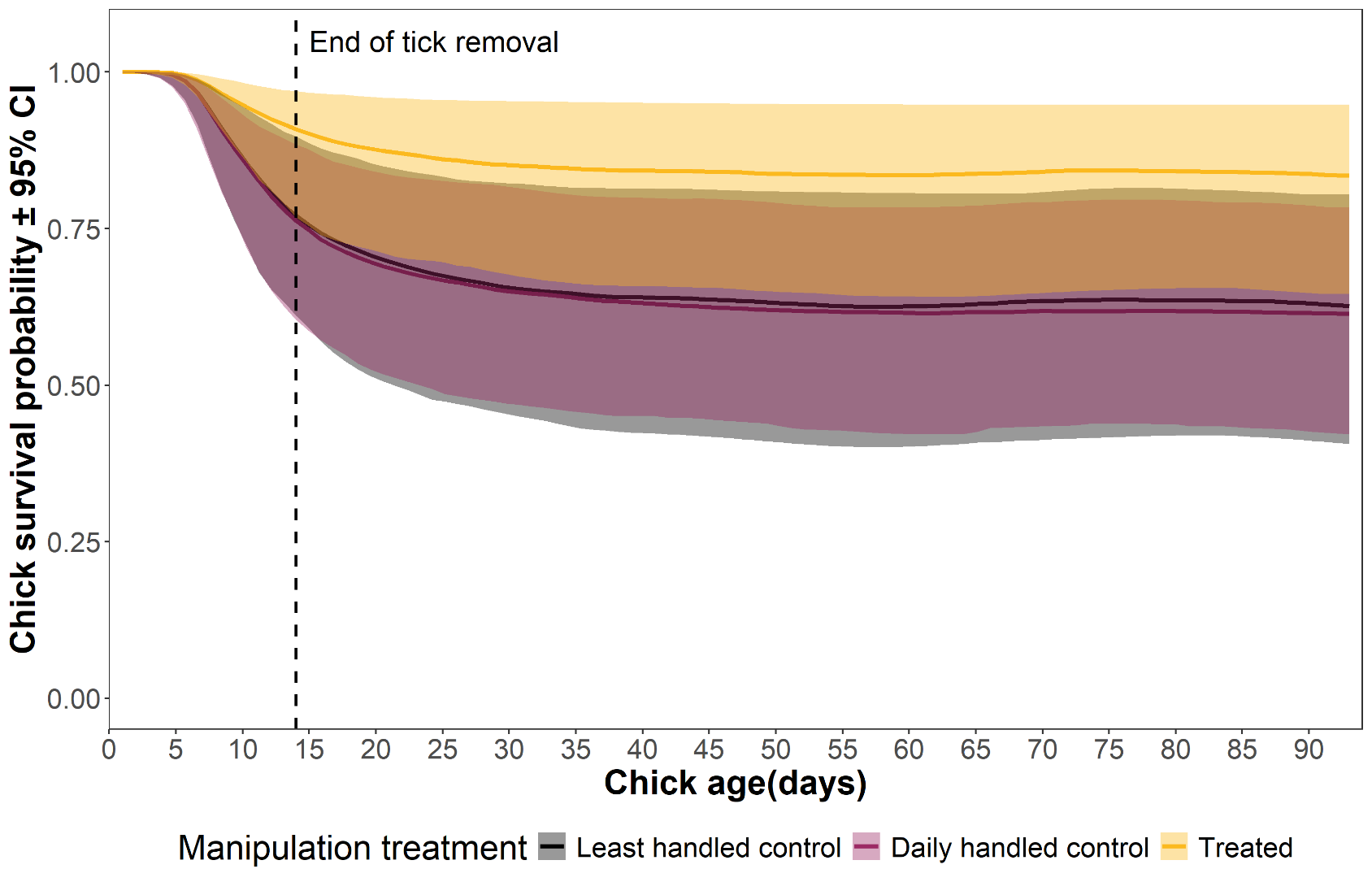


Figure S3 – Survival probability curve of the three manipulation treatment albatross chicks as a function of chick age (in days) throughout the fieldwork period. Solid lines represent the mean survival probability obtained from the Bayesian model with the explanatory variables: manipulation treatment, sub-colony, hatching date and body mass at 5 days old. Shaded areas indicate the 95 % credible interval obtained from that model. The vertical dashed line indicates the age at which the removal of the ticks on the treated chicks ceased (14 days old). The posterior predictive distributions were calculated while holding constant the variables hatching date and body mass at 5 days old (at their mean value) and the sub-colony to C (as this sub-colony had greater sample size than sub-colony B).


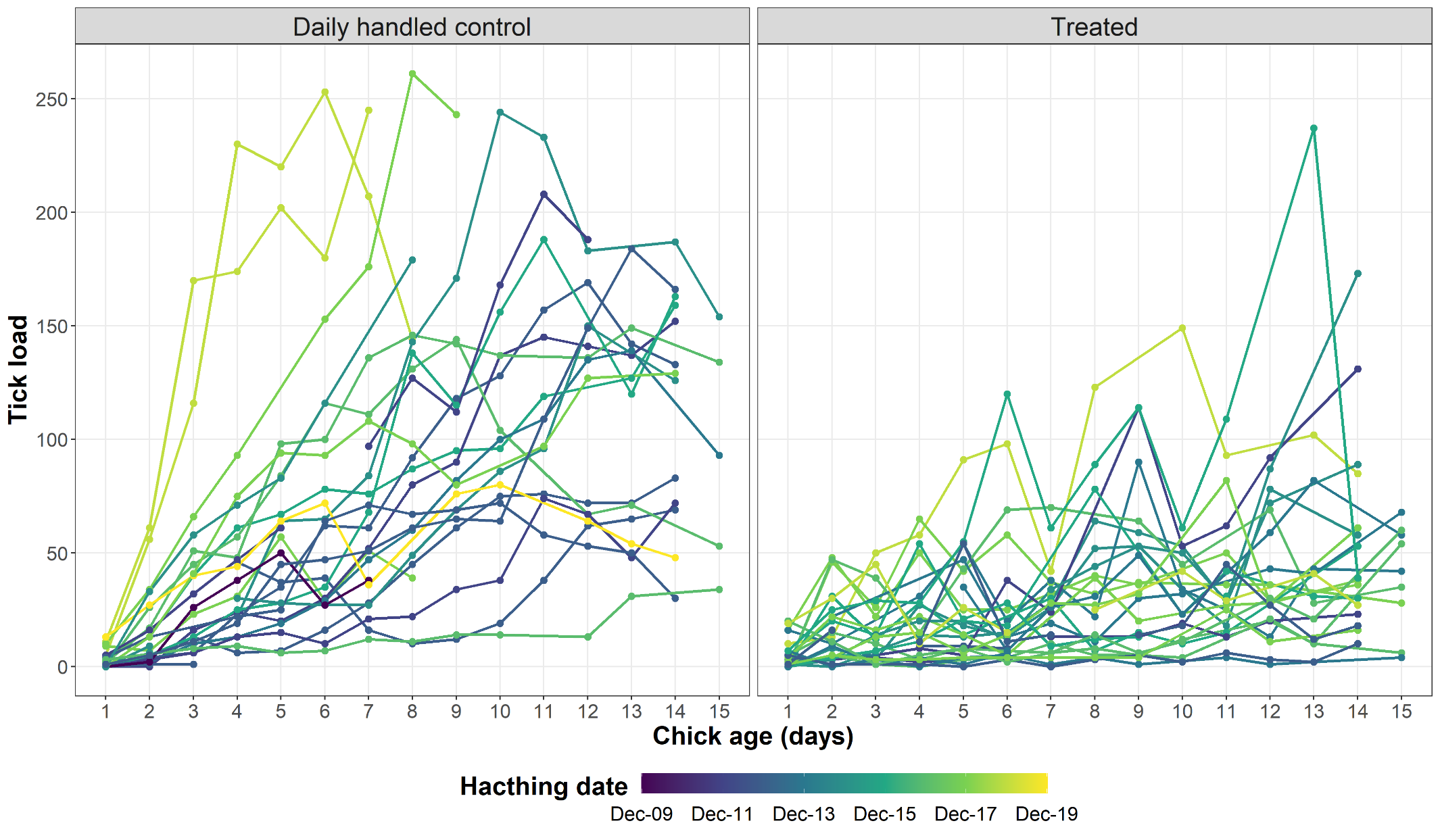


Figure S4 – Tick load (i.e., number of ticks) counted on daily handled control and treated black-browed albatross chicks by age (in days) with different colours by hatching date. Each line corresponds to an individual. Due to adverse weather conditions, there were some days that we could not manipulate the chicks and thus on some chicks the tick load was counted at 15 days old instead of 14 days old.


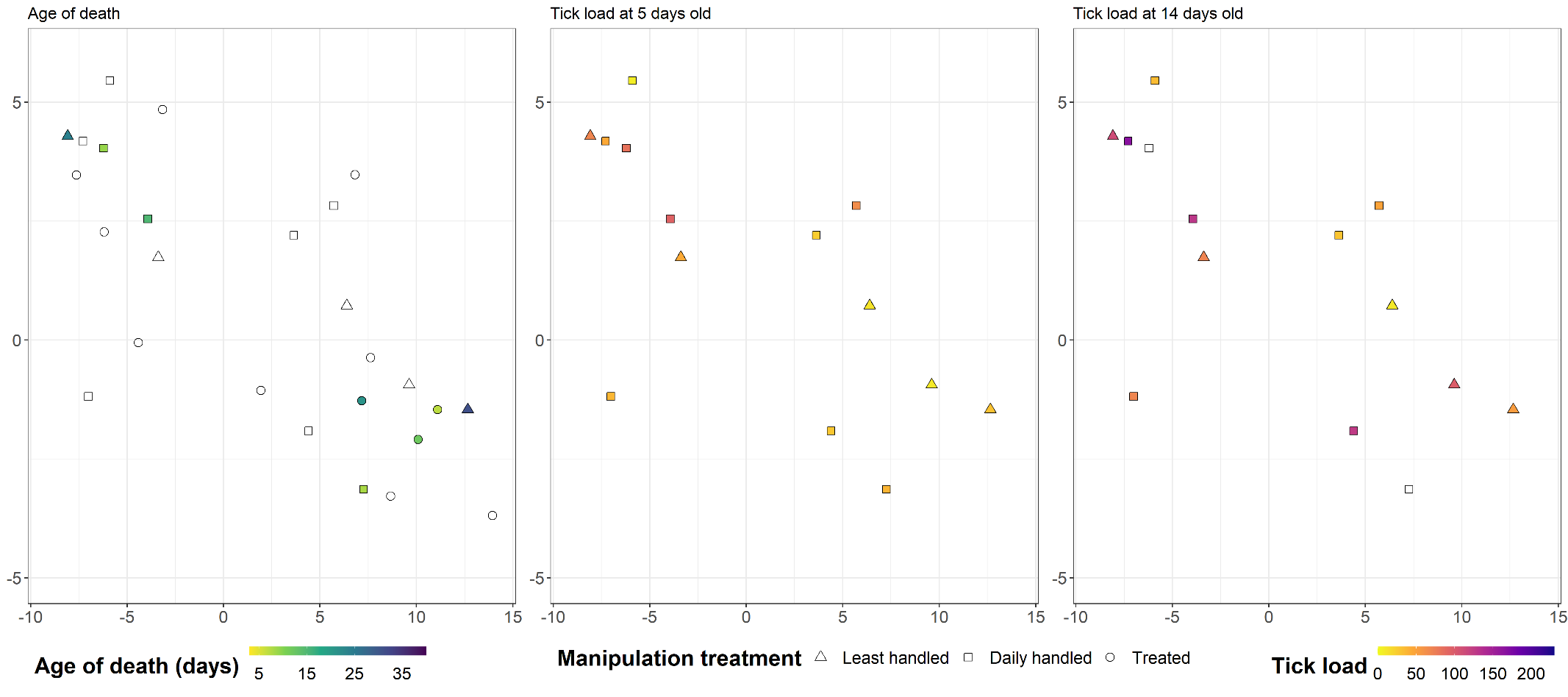


Figure S5 – Representation of the relative positions of the nests of black-browed albatrosses included in this study on sub-colony B based on the distance and angles between the closest nests. Nest type (i.e., treatment group) is represented by different symbol shapes. On the left panel is shown the age of death of the chick: open symbols represent chicks that remained alive until the end of the fieldwork (beginning of March), while the colour gradient represents the age of death of the remaining chicks. On the central and left panels, the colour gradient represents the tick load found on each least handled control and daily handled control chick at 5 and 14 days old, respectively, while open symbols correspond to chicks that died before reaching that age. Axis values represent fictitious coordinates from a nest selected randomly to be positioned at the origin (0,0).


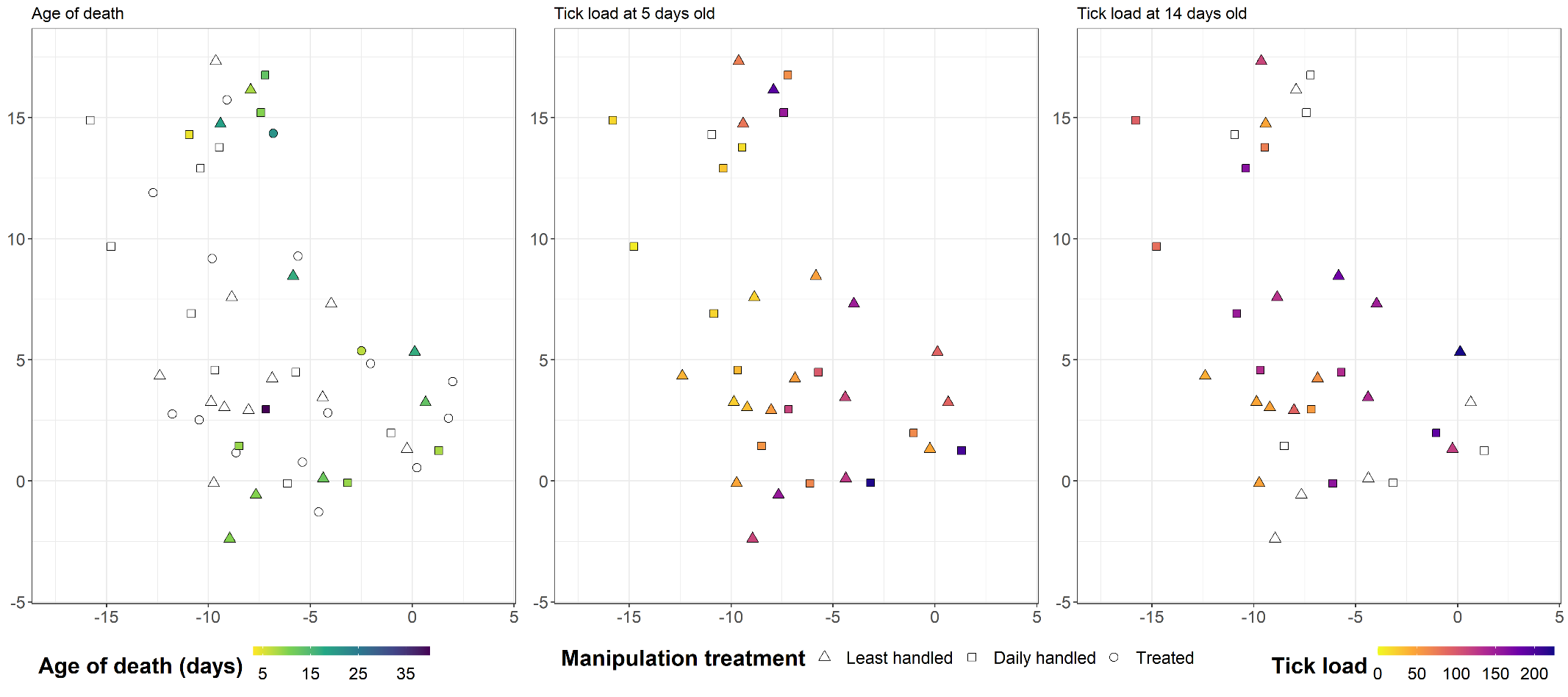


Figure S6 - Representation of the relative positions of the nests of black-browed albatrosses included in this study on sub-colony C based on the distance and angles between the closest nests. Nest type is represented by different symbol shapes. On the left panel is shown the age of death of the chick: open symbols represent chicks that remained alive until the end of the fieldwork (beginning of March), while the colour gradient represents the age of death of the remaining chicks. On the central and left panels, the colour gradient represents the tick load found on least handled control and daily handled control chick at 5 and 14 days old, respectively, while open symbols correspond to chicks that died before reaching that age. Axis values represent fictitious coordinates from a nest selected randomly to be positioned at the origin (0,0).


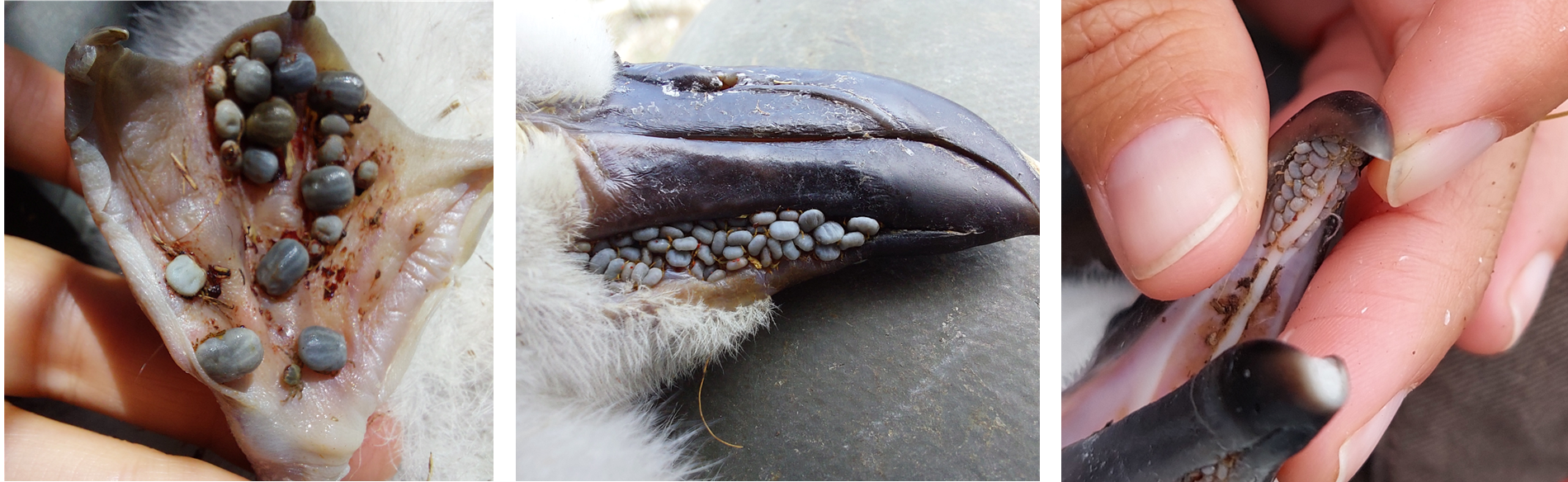


Figure S7 – Photographs of most typical areas where a high tick load was found on the study chicks, i.e., on the foot (left), under the bill (middle) and inside the bill (right).

Table S1 – Regression coefficients estimates (and respective standard error, SE) and 95% credible interval (CI) of the explanatory variables used in the Bayesian linear mixed models (body mass and bill length), linear models (mass gain and bill growth rate) or survival model performed to assess the causal effects of tick removal in treated black-browed albatross chicks in comparison with control chicks (daily handled and least handled control chicks segregated). In all models, all parameters had Ȓ equal to 1.00, indicating good model convergence, and Bulk and Tail Effective Sample Size (ESS) higher than 10,000. Bayes R^2^ is a measure of how well the model explains the variance in the observed data, this metric is not currently available survival models. Credible intervals that do not include 0 are marked in bold. The symbol “:” represents an interaction between model explanatory variables. In the case of the Bayesian Cox model, each coefficient represents the log hazard ratio for a one-unit change in the model explanatory variable.

| **Parameter estimate ± SE [lower, upper 95% CI]** | **Body mass** | **Bill length** | **Daily mass gain between 5 and 14 days old** | **Daily bill growth between 5 and 14 days old** | **Survival rate to 14 days old** | **Survival rate to fledgling** |
| --- | --- | --- | --- | --- | --- | --- |
| Bayes R^2^ | 0.72 ± 0.03  [0.66, 0.77] | 0.88 ± 0.01  [0.85, 0.90] | 0.14 ± 0.07  [0.03, 0.28] | 0.19 ± 0.07  [0.06, 0.33] |  |  |
| Intercept | **284.00 ± 19.94**  **[244.81, 323.24]** | **35.90 ± 0.35**  **[35.21, 36.59]** | **43.06 ± 5.67**  **[31.90, 54.19]** | **1.20 ± 0.07**  **[1.06, 1.35]** | **-12.18 ± 5.68**  **[-25.14, -3.74]** | **-19.62 ± 6.50**  **[-33.47, -8.48]** |
| Sigma | **4.30 ± 0.11**  **[4.05, 4.50]** | 0.21 ± 0.14  [-0.11, 0.45] |  |  |  |  |
| Sigma: age 14 days | **0.98 ± 0.14**  **[0.73, 1.27]** | **0.79 ± 0.16**  **[0.50, 1.14]** |  |  |  |  |
| Age (14 days) | **372.42 ± 50.55**  **[273.41, 472.08]** | **10.34 ± 0.72**  **[8.94, 11.75]** |  |  |  |  |
| Sub-colony (C) | **44.77 ± 18.58**  **[8.32, 81.28]** | 0.09 ± 0.33  [-0.55, 0.73] | -5.46 ± 5.29  [-15.86, 4.95] | -0.13 ± 0.07  [-0.27, 0.01] | 0.95 ± 0.61  [-0.19, 2.21] | 0.36 ± 0.44  [-0.49, 1.24] |
| Hatching date | 12.27 ± 8.52  [-4.50, 28.99] | 0.28 ± 0.15  [-0.01, 0.57] | -0.36 ± 2.58  [-5.43, 4.72] | -0.01 ± 0.03  [-0.07, 0.06] | 0.13 ± 0.29  [-0.45, 0.70] | 0.22 ± 0.24  [-0.25, 0.68] |
| Manipulation treatment (least handled control) | 7.94 ± 23.27  [-37.96, 53.67] | 0.43 ± 0.41  [-0.37, 1.24] | 2.74 ± 6.67  [-10.32, 15.86] | -0.09 ± 0.09  [-0.26, 0.08] | -0.41 ± 0.61  [-1.62, 0.77] | 0.00 ± 0.46  [-0.90, 0.89] |
| Manipulation treatment (treated) | 3.91 ± 22.13  [-39.59, 47.43] | 0.22 ± 0.39  [-0.55, 0.99] | **12.69 ± 6.24**  **[0.44, 24.98]** | 0.09 ± 0.08  [-0.07, 0.25] | -1.05 ± 0.70  [-2.50, 0.26] | -0.99 ± 0.56  [-2.14, 0.07] |
| Manipulation treatment (least handled control):14 days old | 26.88 ± 69.77  [-111.35, 163.00] | -0.73 ± 0.99  [-2.68, 1.20] |  |  |  |  |
| Manipulation treatment (treated):14 days old | **129.32 ± 65.79**  **[0.01, 258.75]** | 1.06 ± 0.94  [-0.78, 2.90] |  |  |  |  |
| Body mass at 5 days old |  |  |  |  | **-0.99 ± 0.31**  **[-1.62, -0.40]** | **-0.80 ± 0.24**  **[-1.27, -0.34]** |

Table S2 – Regression coefficients estimates (and respective standard error, SE) and 95% credible interval (CI) of the explanatory variables used in the Bayesian linear models to compare the chick’s body mass at 59 days old between daily handled control, least handled control and treated chicks and of between both control chick groups together versus treated chicks. In both models, all parameters had Ȓ equal to 1.00, indicating good model convergence, and Bulk and Tail Effective Sample Size (ESS) higher than 10,000. Bayes R^2^ is a measure of how well the model explains the variance in the observed data. Credible intervals that do not include 0 are marked in bold.

| **Parameter estimate ± SE**  **[lower, upper 95% CI]** | **Body mass at 59 days**  **(least handled control vs daily handled control vs treated chicks)** | **Body mass at 59 days**  **(control (both types) vs treated chicks)** |
| --- | --- | --- |
| Bayes R^2^ | 0.14 ± 0.07  [0.03, 0.28] | 0.13 ± 0.07  [0.02, 0.27] |
| Intercept | **3,548.10 ± 168.57**  **[3,217.56, 3,878.71]** | **3,545.67 ± 142.54**  **[3265.24, 3827.51]** |
| Sub-colony (C) | -245.00 ± 152.92  [-546.31, 55.54] | -245.06 ± 149.53  [-539.98, 49.41] |
| Hatching date | -54.58 ± 73.75  [-199.92, 97.72] | -54.61 ± 71.95  [-196.17, 86.78] |
| Manipulation treatment (least handled control) | -5.24 ± 195.55  [-389.02, 381.32] |  |
| Manipulation treatment (treated) | 158.97 ± 177.12  [-190.51, 508.37] | 161.01 ± 144.18  [-123.21, 443.44] |
